# Readily usable strategies to control mastitis for production augmentation in dairy cattle: A review

**DOI:** 10.14202/vetworld.2020.2364-2370

**Published:** 2020-11-09

**Authors:** Champak Bhakat, A. Mohammad, D. K. Mandal, A. Mandal, S. Rai, A. Chatterjee, M. K. Ghosh, T. K. Dutta

**Affiliations:** 1 Department of Livestock Production and Management, ICAR-National Dairy Research Institute, ERS, Kalyani Nadia, West Bengal, India; 2 Department of Dairy Extension, ICAR-National Dairy Research Institute, ERS, Kalyani Nadia, West Bengal, India; 3 Department of Animal Breeding, ICAR-National Dairy Research Institute, ERS, Kalyani Nadia, West Bengal, India; 4 Department of Animal Nutrition, ICAR-National Dairy Research Institute, ERS, Kalyani Nadia, West Bengal, India

**Keywords:** dairy cattle, hygiene, mastitis, milk production, pathogen, strategies

## Abstract

Mastitis in dairy cattle is the most common management disorder that causes higher economic losses by lowering production and quality of milk leads to substantial economical loss. The aim of this article was to review worldwide important advances in strategies to control mastitis for production augmentation in dairy cattle. Many scientists worked to identify effective strategies to control mastitis caused by *Streptococcus agalactiae, Staphylococcus aureus*, and others. It is necessary to identify mechanisms of infection, define clinical and subclinical states of disease, determine exposure time, and identify pathogen-specific characteristics. Evolvement of management strategies that incorporated hygienic procedures (animal, floor, and milkman), post milking standing period of animal and strategic use of antibiotic or herbal therapy at dry-off, nutritional supplementation, fly control, body condition score optimization, etc., resulted in widespread control of mastitis. The udder, teat of animal, scientific management of milking, automatic milking procedure, genetic selection are considered as important factors to control mastitis. As farm management changed, scientists were directed to redefine control of mastitis caused by opportunistic pathogens of environmental sources and have sought to explore management strategies which will maintain animal well-being in a judicial way. Although significant advances in mastitis management have been made changing herd structure, changing climatic scenario and more rigorous milk processing standards ensure that mastitis will remain important issue for future research.

## Introduction

Mastitis is defined as inflammation of parenchyma of mammary glands and is characterized by physical, chemical, and usually bacteriological changes in milk and pathological changes in glandular tissues [1].

The significance of study focus on that the global problem of mastitis which is multifactorial disease but lacked research that allowed prioritizing effect of different strategies to control it. The treatment of mastitis would not be the only solution and called for research to define the value of various unproven management practices. In India (tropics), annual economic loss due to mastitis was reported INR 60532.1 million, where the majority was found due to sub-clinical mastitis (70-80%) which accounted around INR 43653.2 million [2].

The aim of this article was to review worldwide important advances in strategies to control mastitis for production augmentation in dairy cattle.

## Etiological Factors and Common Pathogen

Some studies have been reported that the incidence of sub-clinical mastitis ranged from 19.20 to 83% in cows [2]. For decades, *Streptococcus agalactiae* and *Staphylococcus aureus* were considered the most important etiology. Researchers reported a three-phase process for development of mastitis, namely, (i) invasion of an organism (with or without infection), (ii) infection (bacteria became established in udder), and (iii) inflammation. This process continues to serve as basis of our understanding of mastitis. Etiological factors such as host (breed, high yield, age, parity, milking interval, milk and somatic cell count [SCC], stage of lactation, udder defense mechanism, udder conformation, dry period, teat injuries, and genetic resistance), pathogen (virulence factor, and number of organisms), blind treatment, management practices (udder hygiene, poor teat condition, poor environmental hygiene, sanitation, large herd size, use of hand wash, improper teat dipping, milking technique, and milking machine), and nutrition (Vitamin E, and Selenium deficiency) among others have been reported to be important in the prevalence of mastitis [3]. Although numerous bacteria are identified as able to cause intra-mammary infection (IMI), initial emphasis of mastitis control was directed at pathogens that were known to spread among cows in a contagious manner when teats were exposed to bacteria in milk that originated from an infected mammary gland. Sub-clinical mastitis is 15-40 times more prevalent than clinical mastitis [4]. In a report by Kumar *et al*. [5], *Streptococcus dysgalactiae* was major (50.00%) organism isolated from cases of sub-clinical mastitis in cows, followed by *S. aureus* and others. Kumar *et al*. [5] also noted that while >20 types of infections can cause mastitis; at least 99% are caused by *S. agalactiae*, other streptococci, staphylococci, and bacillary mastitis (including coliform and pseudomonas).

Research Committee of National Mastitis Council of India [3] reported about growth requirements of various coliform bacteria, mechanisms of IMI (with emphasis on exposure and movement through teat canal), an explanation of pathogenesis (including recognition that magnitude of inflammation is dependent on host factors), an excellent portrayal of epidemiology and risk factors with recommendations for a model control program. Sharma *et al*. [3] reported many other organisms including *Trueperella pyogenes, Pseudomonas aeruginosa*, and *Clostridium perfringens* and others such as *Mycobacterium, Mycoplasma, Pasteurella* species, and yeasts. Differences among pathogens, the importance of IMI during dry period, the high rate of spontaneous clearance of Gram-negative IMI, and increased rate of clinical cases (vs. sub-clinical) associated with environmental pathogens were all thoroughly described. They correctly predicted challenges of reducing environmental mastitis in herds that have effectively controlled contagious organisms and summarized recommendations for mastitis control that remains relevant for modern intensively managed dairy farms.

## Strategies to Control Mastitis in Dairy Cattle

The salient research finding of various strategies related to heifer’s mastitis (Table-1) [6-10], dry cow management (Table-2) [11-19], and lactating cow’s mastitis management strategies (Table-3) [20-30]. are presented in tabular form. Control of mastitis and maintaining udder health in manual and automatic milking systems remains a challenge. Role of competent attendants in managing udder health remains as important today as in past decades [31]. As milking machines became popular, describing appropriate milking procedures and following management strategies are important priorities.

**Table-1:** Effective strategies to control heifer’s mastitis [6-10].

Sl. no.	Researchers	Year	Salient findings
1.	Singh *et al*.	2020	At lower Gangetic regional village level Jersey crossbred heifer is susceptible to udder problem [6]
2.	Salvador *et al*.	2014	Define prevalence and control of IMI in dairy heifers. Although the milk appears normal, heifer with subclinical IMI produce less milk and with compromised quality [7]
3.	Trinidad *et al*.	1990	A high prevalence of IMI caused by *Staphylococcus aureus* was initially reported for prepartum heifers in the southern United States [8]
4.	Iraguha *et al*.	2015	Subclinical mastitis can lead to a 10-20% decrease in milk production. In addition, it has an undesirable effect on the constituents and nutritional value of milk, rendering it of low quality, and less fit for processing [9]
5.	De Vliegher *et al.*	2012	Although heifers have a relatively low prevalence of infection with major pathogens, many are colonized by CNS [10]

**Table-2:** Dry cow management strategies [11-19].

Sl. no.	Researchers	Year	Salient findings
1.	Singh *et al.*	2020	Find out that alteration of dry period feeding management can improve dry cow BCS, thus lead to lower NEB, milk SCC, and maintain better udder health [11]
2.	Bhakat *et al.*	2019	Repetition of mastitis occurrence is a major constraint for dairy development in hot humid tropical regions [12]
3.	Bhakat *et al.*	2016	The Log _10_ SCC (cells/ml) was significantly (p<0.01) higher in IMI cows (6.55±0.05) as compared to no-IMI Jersey crossbred cows (4.05±0.04) at hot humid tropic [13]
4.	Kumari *et al.*	2019	Dry cow therapy (intra-mammary) using various herbal preparation with internal and external teat sealant can be alternate management practices to control mastitis during post-calving period and also concluded that herbal fly control measures significantly (p<0.01) reduced sub-clinical mastitis cases [14]
5.	Hillerton and Kliem	2002	Developed and introduced a commercially non-antibiotic internal teat sealant [15]
6.	Cameron *et al.* Scherpenzeel *et al.* Halasa *et al.*	2014 2014 2010	The continued decline of IMI caused by *Streptococcus agalactiae* and *Staphylococcus aureus* and availability of a non-antibiotic alternative to prevent new IMI [16-18]
7.	Huijps and Hogeveen	2007	Economic models have demonstrated that decision to use either selective or comprehensive antibiotic DCT has highly farm specific [19]

**Table-3:** Lactating cow’s mastitis management strategies [20-30].

Sl. no.	Researchers	Year	Salient findings
1.	Paul *et al*.	2020	Optimization of BCS at calving time can improve udder health status in lactating cows [20]
2.	Barkema *et al.*	2006	Emphasized that the prognosis for treatment of *Streptococcus agalactiae* was partial because of location of infection in milk duct system. In contrast, when referring to *Staphylococcus aureus*, they reported that the prognosis regarding therapy is disappointingly low because organisms penetrate the duct walls of udder and become established in several foci [21]
3.	Barkema *et al.*	2006	Find out about cow, pathogen and treatment factors that contribute to therapeutic success of cows infected with *Staphylococcus aureus* again emphasized that only selected animals will respond to antibiotic therapy [21]
4.	Singh *et al.*	2020	The pre and postpartum alpha-tocopherol supplementation improved udder health status, milk yield, body condition score of lactating cows at tropical lower Gangetic region [22]
5.	Lago *et al*. Lago *et al*.	2011a 2011b	The increased proportion of culture-negative clinical cases and increased diversity of etiological agents have encouraged development of selective treatment protocols [23,24]
6.	Ruegg	2017	Recommended for treatment of clinical mastitis are based on targeted antibiotic usage for most Gram-positive cases while allowing time for spontaneous cure of most other cases [25]
7.	Kuipers *et al* Stevens *et al*. Saini *et al.*	2016 2016 2012	Mastitis remains mostly common bacterial disease in maximum dairy farms and consequently, mastitis treatment and prevention account for majority of antimicrobials administered to adult dairy cows [26-28]
8.	Kumari *et al*.	2018	Find out that supplementation of trisodium citrate (at 10 mg/kg bw) to lactating cow can reduce the occurrences of subclinical mastitis [29]
9.	Kumari *et al*.	2020	Adoption of scientific management practices such as full hand milking procedure, post-milking standing period of 35 min standing of cow, and increased hygiene (cow, milkers, and shed) status of lactating cow can significantly reduce the occurrences of subclinical mastitis [30]

## Role of Udder, Teat, and Scientific Management of Milking Procedure to Control Mastitis

Bharti *et al*. [32] reported that rate of incidence of sub-clinical mastitis was significantly (p<0.01) higher in hind than front quarters and therefore, hind quarter required more attention during different udder health management programs. Role of association between bacterial colonization on teat surface and development of IMI has been found out. Bharti *et al*. [33] concluded that the shape of udder type and teat end type significantly (p<0.01) contributed to IMI and sub-clinical mastitis in Jersey crossbred cows. The use of management practices which reduce bacterial contamination of teat ends is a basic aspect of mastitis control. Scientists established the importance of post-milking teat disinfection for control of contagious microbes. Bharti *et al*. [33] reported that post-milking teat antiseptic was regarded as the single most effective practice to control IMI of lactating cow but cautioned that it was not equally effective against coliforms and many streptococci. Pre-milking sanitation had usually been performed by washing udders, teats, pre-dipping, and post-dipping with water or disinfectants, but demonstrated that pre-milking disinfection of teats, followed by effective drying dramatically reduced development of IMI caused by *Streptococcus uberis*. Bhakat *et al*. [34] reported effective field management practices such as pre-dipping and post-dipping (udder washing before and after) each milking, full hand milking, family labor involvement, and daily unlimited supply of drinking water provision to animal can reduce sub-clinical mastitis with improvement of udder health and milk quality in tropical region. Bhakat *et al*. [34] recommended standardization of pre-milking procedures and proper udder hygiene at each milking. It is milk processor preferences and requirement for milk with little bacterial contamination, sediment, or residues which will continue to encourage adoption of increasing strict teat preparation practices. Bhakat *et al*. [34] had studied physiological mechanisms of milk secretion and ejection, and hormone like oxytocin was identified as a substance that could positively stimulate milk flow. As milking machines were adopted, factors that could influence milk ejection were studied. It was also demonstrated that fear had a significant effect on reducing milk ejection. This was a potentially important finding because incomplete milking of cows chronically infected with *S. agalactiae* which was responsible for occurrence of clinical mastitis [35].

## Role of Automatic Milking Procedure to Control Mastitis

Bharti *et al*. [36] reported that IMI and incidence of sub-clinical mastitis in machine milked cows caused considerable changes in milk SCC, milk yield, and pH of milk. Milk samples obtained from such infected animals had lower test day milk yield, fat%, and solid not fat% but higher SCC and milk pH. The effects of vacuum level, vacuum stability, and milking duration on risk of mastitis were identified. Baxter *et al*. [37] found that both vacuum fluctuations and milking duration should be minimized to reduce the risk of new IMI associated with liner slips. As milking machines rapidly replaced hand milking, researchers became concerned that the machines could cause irritation and serve as fomites for spreading mastitis among cows. Research is needed to determine how milking machines functioned relative to physiology of milk secretion and how adoption of machine milking would influence the occurrence of mastitis.

Bhakat *et al*. [38] reported that IMI can be reduced in machine milking practices in comparison to hand milking practices with higher milk production but without affecting milk quality in Jersey crossbred cows at tropical lower Gangetic region. Thompson *et al*. [39] reported that the increasing role for automation in milking process and automatic detacher had been the most important development in milking automation, predicted that sensors would be developed that would result in further automation not only of milking tasks but also of management of data recording and analysis. Later on, automatic milking systems have become common in many regions but effective use of data from systems is still not optimized [40]. By this time, many herds had controlled *S. agalactiae* and *S. aureus* and prevalence of IMI had declined. Advances in milking machines had greatly improved vacuum stability and installation standards for milking systems had been developed. Scientists reported that milking machine could influence new IMI by serving as a fomite, allowing cross-infections within cows, and damaging teat sphincters. They demonstrated that only 6.6% of new IMI were accounted by milking machine and concluded that there was no convincing evidence linking the machine milking to overall prevalence of herd infection. Bhakat *et al*. [41] reported that defective floor type of milking shed/parlor can affect udder health of dairy cattle adversely.

## Role of Body Condition Score (BCS) and Genetic Selection Procedure to Control Mastitis

Paul and Bhakat [42] found that higher and lower BCS at calving significantly (p<0.05) increase SCC in milk. Paul *et al*. [43] standardized BCS technique for Jersey crossbred cow and concluded that this technique of BCS at calving can be used as a reliable criterion in selecting Jersey crossbred cows for higher milk production with better udder health status at organized farm in tropical India. Recently, Paul *et al*. [20] reported scientific procedure to conduct BCS regularly of dairy animals to maintain udder health status effectively. Singh *et al*. [44] found correlation among optimum BCS of dry and lactating cows with better udder health maintenance by lowering subclinical mastitis. It was observed differences in rate of IMI among separate cow families of equal productivity within a single farm and noted that heritable differences in susceptibility may contribute for development of IMI. The ability to use genetic selection to reduce mastitis has gradually evolved. Early estimates of heritability of mastitis ranged from 0.27 to 0.38. Selection for mastitis resistance was encouraged because genetics increases in milk yield were shown to be correlated with increased susceptibility to mastitis [45]. Somatic cell scores were incorporated into US selection indices in 1994. Although improving mastitis resistance had not been the highest priority of US dairy farmers, considerable progress has occurred in other countries [46] and future innovations in genomic selection technologies will likely be used to accelerate genetic gains in resistance to mastitis. Until widespread adoption of SCC in DHI programs, advancements in genetic selection for mastitis resistance were not possible.

## Role of Hygiene and Nutritional Strategies and Animal Behavior to Control Mastitis

Paul *et al*. [47] had been seen that higher SCC due to poor hygiene practices was very critical because more influx of milk SCC not only disrupts the mammary epithelium but also decreases milk quality which, in turn, leads to the lower returns. Bhakat *et al*. [48] found that farmer of hot-humid tropics having more than 3 cows, most of them (50%) were maintaining poor hygiene status, cleanliness in their animal, shed, and milkman which were vulnerable factors for sub-clinical mastitis in dairy cows.n This institute (Eastern Regional Station-National Dairy Research Institute [NDRI]) adopted village with most of the small category farmers maintaining 1-3 dairy cows [49]. It is also essential to understand the important risk factors associated with nutritional management for incidence of sub-clinical mastitis in dairy cattle. It does not create visible changes in milk or in udder [50]. It was erroneously suggested that feeding high concentrate diets was a risk factor for mastitis but direct effects of nutrition on mastitis were not reported until performed experiments that demonstrated that dietary deficiencies of selenium and Vitamin E increased incidence and duration of clinical mastitis. Initial experiments were supported by later field studies that demonstrated increased sub-clinical and clinical mastitis in selenium-deficient farms. Kumari *et al*. [51] found that supplementation of tri-sodium citrate to lactating cow was an effective, easy, and cost-effective management practices which will support farmers in raising their income by significantly (p<0.01) lowering sub-clinical mastitis in tropical region. Wathore and Bhakat [52] found in a farm experimentation that the Vit-E (feed grade) supplementation during 30 days pre-partum and 60 days post-partum, provided positive and significant (p<0.01) results to the lower down milk SCC and incidence of sub-clinical mastitis. Singh *et al*. [53] tested the management strategies of alpha-tocopherol supplementation at field condition which significantly improved udder health status of Jersey crossbred cows.

Bharti and Bhakat [54] reported that post-milking standing period of 35 min by provision of fresh feed/fodder immediately after each milking can significantly (p<0.01) reduce sub-clinical mastitis in Jersey crossbred cows at tropics. Mastitis is caused by a variety of bacterial pathogens with different strains that vary among farms and over time. Although vaccines have been used to effectively control other bacterial diseases of dairy cows, the nature of mastitis poses many challenges to their success. The development of effective vaccines to protect cows from developing new IMI has been a goal of many mastitis workers. The site of IMI within mammary gland, virulence characteristics, and immunogenic capabilities all vary among pathogens. Bhakat *et al*. [55] reported that the IMI lead to changes in glandular tissue of the udder and it was essential to monitor IMI in dairy cows to maintain milk quality and udder health.

## Conclusion

This review highlights the worldwide important advances in strategies to control mastitis for production augmentation in dairy cattle. Role of udder, teat of animal, scientific management of milking procedure, automatic milking procedure, BCS, genetic selection procedure, hygiene, nutritional aspects, animal behavior, etc., is considered to control mastitis. There is a need to provide infrastructure, necessary facilities and training to help farmers to efficiently adopt proven strategies that minimize occurrences of mastitis which result in production of higher quantity and quality of milk and continued advances in management and control of mastitis are very much necessary to ensure sustainability of dairy farming.

## Authors’ Contributions

CB conceptualized and drafted the review, prepared, and edited the manuscript. AM, DKM, AMa, SR, and AC collected literatures. MKG and TKD edited and finalized the manuscript. All authors read and approved the final manuscript.
